# First Experience Using the Sheathless Hyperion Guiding Catheter System Designed for Direct Insertion

**DOI:** 10.1155/2022/5668728

**Published:** 2022-02-22

**Authors:** Andrew Borrie, Sarah Fairley, Scott A. Harding

**Affiliations:** Wellington Hospital, Wellington, New Zealand

## Abstract

**Background:**

Use of sheathless guiding catheters for transradial PCI has the potential to reduce radial trauma and allow use of larger catheters to facilitate complex PCI. The new sheathless Hyperion guide catheter (SHGC) system allows direct insertion of the SHGC using a 20G needle or IV cannula, a 0.025″ Silverway wire, and a dilator. We report the first clinical experience.

**Methods:**

We prospectively evaluated outcomes in consecutive patients undergoing PCI using radial access and the SHGC catheter at our institution between June 2020 and June 2021. There were no exclusion criteria.

**Results:**

The study included 120 patients, mean age 67 ± 12.6 years, 79.2% male. Insertion of a SHGC was attempted in 128 radial arteries and was successful in all cases. The SHGC was inserted directly in 74 (57.8%), following initial sheath removal in 24 (20.5%) and through the initial sheath in 30 (26.2%). Coronary artery engagement with a SHGC was successful in 126 (98.4%). A total of 150 lesions were treated, the majority being complex: 16.1% chronic total occlusions, 37.1% calcified, 30.6% bifurcation, and 43.5% long lesions. Angiographic success was achieved in 149 (99.2%) lesions. Periprocedural myocardial infarction occurred in 5 (4.2%) patients. There was no in-hospital urgent revascularisation or death and no major bleeding or vascular complications. Occlusion occurred in 2 (1.6%) radial arteries.

**Conclusion:**

This first clinical experience with the SHGC demonstrates that direct insertion is safe and effective and that the use of the SHGC allows complex interventions to be undertaken transradially with a high success rate.

## 1. Introduction

Transradial access (TRA) has been increasingly used worldwide for percutaneous coronary intervention (PCI) and has become the standard in many countries. This change has been driven by a large body of research demonstrating that TRA is associated with a reduced risk of access site bleeding, vascular complications, and may reduce mortality in those with acute coronary syndromes (ACS) when compared to transfemoral access [1–5]. Recent American and European guidelines have endorsed radial as the preferred route of access for coronary diagnostic and interventional procedures [6, 7].

Some operators have been reluctant to perform complex coronary intervention using TRA due to perceived limitations. The maximum size of the guiding catheter that can be used for TRPCI is limited by the relatively small inner lumen of the radial artery [8]. This potentially limits backup support and the types of adjunctive devices and procedures that can be performed via TRA. A further issue is radial artery occlusion, the most frequent postprocedure complication of TRA. This is important as it prevents ipsilateral TRA for future procedures. The sheath-to-artery diameter ratio has been shown to be an important predictor of radial artery occlusion with occlusion rates increasing with large sheath sizes [8, 9]. Therefore, strategies that facilitate the use of transradial for complex PCI while maintaining radial artery patency are needed.

A sheathless guide catheter can potentially overcome these issues. Traditionally, an introducer sheath which has an outer diameter that is nearly 2F larger than that of the corresponding guiding catheter has been used for TRPCI. Using a guiding catheter that removes the need for an introducer sheath allows the use of larger lumen guiding catheters and may reduce trauma to the radial artery. The sheathless Hyperion guide catheter (SHGC, Asahi Intecc, Aichi, Japan) has been designed with this in mind, maximizing the internal diameter and guide support while minimising outer diameter and trauma to the radial artery. It is unique in that it has been designed for direct insertion without the need for initial sheath placement. We report the first clinical experience with the SHGC and direct insertion.

## 2. Methods

### 2.1. Study Design and Population

This was a physician initiated single centre prospective observational study. We prospectively evaluated outcomes in consecutive patients who underwent PCI using radial access and the SHGC at our institution between June 2020 and May 2021. Apart from the use of femoral access, there were no exclusion criteria. Use of the SHGC was the operators' discretion. The SHGC was often selected for complex planned PCI when a 7F guiding catheter was preferred or in ad-hoc PCI when the size of the introducer sheath in place was smaller than the guide catheter selected.

### 2.2. Equipment

**Figure 1:**
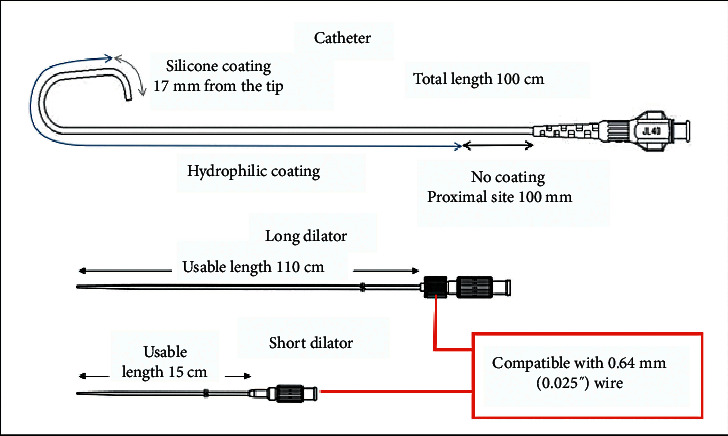
Components of the sheathless Hyperion guide catheter system.

**Figure 2:**
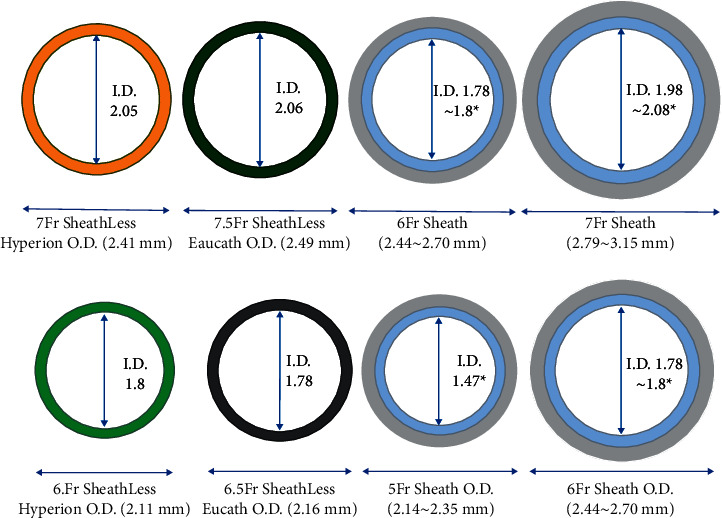
Comparison of sheathless guide catheters and sheaths.  ^*∗*^Internal diameter relating to the internal diameter of the guide catheter within the sheath. I. D., internal diameter; O. D, outer diameter.

The SHGC system (Figure 1) has been designed to allow direct insertion using either a 20G needle or a 20G IV cannula, along with an 0.025″ Silverway wire (Asahi Intecc) and a short dilator (15 cm). There is a long dilator (110 cm) which is radiopaque and is placed within the SHGC for insertion. The inner diameter of the 6F SHGC is 1.8 mm with an external diameter of 2.11 mm, while the inner diameter of the 7F SHGC is 2.05 mm with an external diameter of 2.41 mm (Figure 2). The SHGC has a hydrophilic coating to enhance delivery and uses the same technology as the hyperion guide catheter (Asahi Intecc) including Hyper Shaft and Henka Braid to enhance back up support.

### 2.3. Procedure

**Figure 3:**
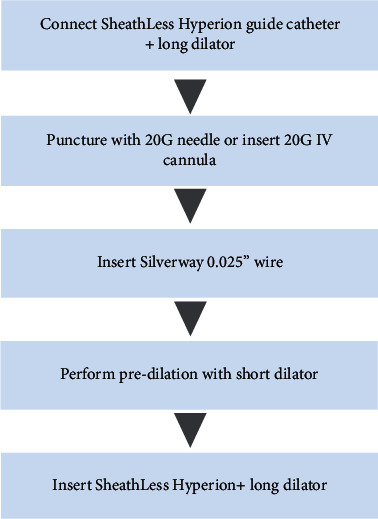
Steps for direct insertion of the sheathless Hyperion guide catheter.

**Figure 4:**
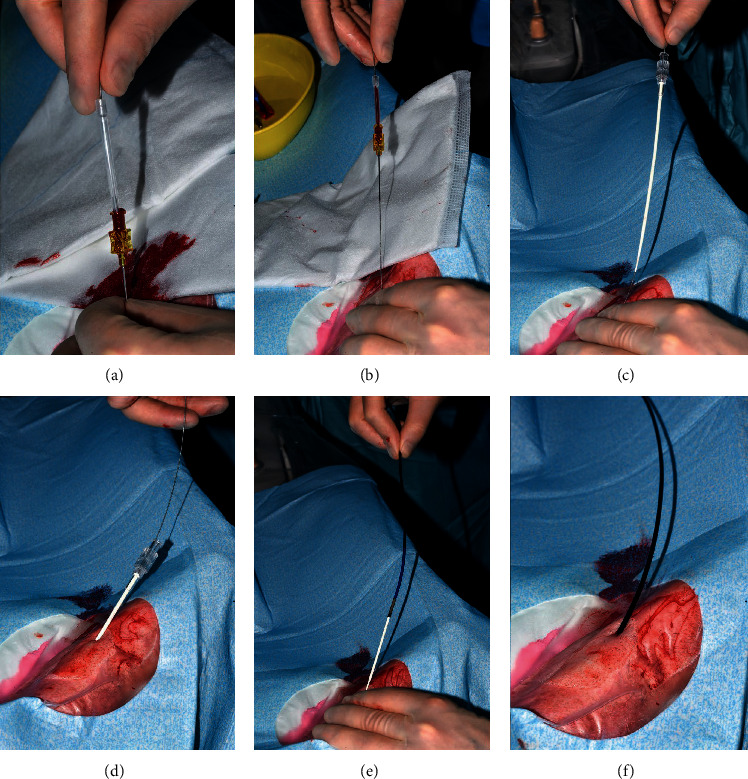
Direct sheathless Hyperion guide catheter insertion. (a) Insertion of a 0.025″ Silverway wire following puncture of the radial artery with a 20G needle. (b) Removal of the 20G needle. (c) Insertion of the short dilator into the radial artery over the 0.025″ Silverway wire. (d) Short dilator in the radial artery. (e) Following removal of the short dilator, the sheathless Hyperion guide catheter with the long dilator inserted is advanced over the 0.025″ Silverway wire. (f) Sheathless Hyperion guide catheter in place in the radial artery.

For direct insertion (Figures 3 and 4), access to the radial artery was gained using either an anterior wall puncture with a 20G needle or the double wall puncture technique using a 20G IV cannula. A 0.025″ angled Silverway wire was then advanced through the needle or IV cannula into the radial artery. Predilation of the skin and tissue was performed with the short dilator. Following this, a SHGC catheter with an appropriate curve with the long dilator inserted was passed over the 0.025″ Silverway wire into the ascending aorta before the long dilator and Silverway wire was removed and the coronary artery engaged in the routine manner.

In cases where ad-hoc PCI was performed using the SHGC, there was already a radial sheath in place from the diagnostic study. If the French size of the selected SHGC was larger than the radial sheath in situ, a 300 cm J-shaped 0.025″ Silverway wire was inserted and the radial sheath was removed. An appropriately shaped SHGC with the long dilator in place was then advanced into the radial artery over the wire without predilation. In cases where the selected French size of the SHGC could be accommodated by the sheath in situ, a 300 cm J-shaped 0.025″ Silverway wire was inserted and an appropriately shaped SHGC with the long dilator in place was inserted through the radial sheath.

In the case of CTO PCI, additional access, access site, and the type of the guide catheter used were at the discretion of the operator. Following direct insertion, patients were given unfractionated heparin at a dose of 100 IU/kg with the aim of maintaining an activated clotting time of >250 s during PCI and >300 s during CTO PCI. Adjunctive boluses of heparin were given during PCI if needed to achieve an activated clotting time target range. For the cases in which initial insertion of 5 or 6F Glidesheath slender (Terumo, Tokyo, Japan) was performed for diagnostic angiography prior to ad-hoc PCI, 5000 IU of unfractionated heparin was given immediately following sheath insertion. A further bolus of unfractionated heparin was given to achieve the targeted activated clotting time following SHGC insertion for the ad-hoc PCI.

The SHGC was removed immediately following the procedure, and haemostasis was achieved by the use of a TR band (Terumo) using a patent haemostasis protocol. Patients routinely had radial artery patency examined clinically. Patients routinely had radial artery patency examined with the reverse Barbeau test. An electrocardiogram and myocardial enzymes were measured prior to discharge.

### 2.4. Study Endpoints and Definitions

The primary endpoint of the study was successful engagement of the target coronary artery with a SHGC from the radial artery. Secondary outcomes included successful completion of the PCI to the target lesion(s) using the SHGC, angiographic success, in-hospital major adverse cardiac events (MACE), procedural success, major bleeding, vascular complications, and radial artery occlusion. Angiographic success was defined as residual stenosis less than 30% with thrombolysis in myocardial infarction (TIMI) grade 3 flow. MACE was defined as a combined endpoint of cardiac death, myocardial infarction (MI), or target lesion revascularisation (TVR). Periprocedural MI was evaluated using both the 4^th^ universal definition [10] and the Society for Cardiovascular Angiography and Interventions (SCAI) definition [11]. Target vessel revascularisation (TVR) was defined as the requirement for either CABG or repeat PCI to the index artery. Procedural success was defined as angiographic success with no in-hospital MACE. Major bleeding was defined as Bleeding Academic Research Consortium (BARC) 3 or 5 bleeding [12]. Significant spasm was defined as grades 2–4 radial spasm as previously defined by Goldsmit et al. [13].

Categorical variables are presented as numbers and percentages. Continuous variables are presented as mean ± standard deviation.

## 3. Results

The study included 120 patients, mean age 67 ± 12.6 years, 79.2% male and 45.8% with acute coronary syndromes. Patient demographics are given in Table 1. Insertion of a sheathless Hyperion catheter was attempted in 128 radial arteries with 8 patients having dual radial access with a SHGC for treatment of chronic total occlusions.

Direct insertion of a SHGC into the radial artery was attempted for 74 (57.8%) and was successful in all cases. In 59, direct access was achieved using anterior wall puncture with a 20G needle and a 0.025″ angled Silverway wire. In the other 15 cases, direct access was achieved using a double wall puncture technique and 20G IV cannula with a 0.025″ angled or J shaped Silverway wire. The short dilator was used for predilation, in all cases of direct insertion, prior to advancement of the SHGC with the long dilator inserted. No skin incision was required. For the remaining catheters, 24 (20.5%) were inserted following initial sheath removal and 30 (26.2%) through the initial sheath. Of the SHGC used, 78 (60.9%) were 7F and 50 (39.1%) were 6F. The shapes of the SHGC used are given in Table 2.

The sheathless Hyperion passed successfully into the ascending aorta in all cases. The primary study endpoint of successful engagement of the target coronary artery by the sheathless Hyperion catheter was achieved in 126 (98.4%). In 6 cases (4.8%), the curve size of the sheathless Hyperion needed to be downsized before engagement could be achieved. In 2 cases (1.6%), the coronary artery could not be engaged due to marked tortuosity of the brachiocephalic artery, and conversion to femoral access was required. There were no cases of significant spasm.

A total of 150 lesions were treated. The majority of lesions were complex (Table 3): 16.7% CTO, 34.7% with moderate or severe calcification, 45.3% long lesions, and 30% bifurcation lesions. Use of adjunctive devices was frequent. Angiographic success was achieved in 149 (99.2%) lesions. In one case, the guide wire failed to cross the CTO. Periprocedural myocardial infarction as defined by the 4^th^ universal definition occurred in 5 (4.2%) patients, but in no patients if the SCAI definition of periprocedural myocardial infarction was applied. There was no in-hospital urgent revascularisation or death. There was no major bleeding or vascular complications and 2 (1.6%) radial artery occlusions.

## 4. Discussion

This is the first study to report use of the SHGC and direct introduction system. The main findings of this study are as follows: the system for direct insertion was very effective with successful insertion in all cases attempted, the target coronary could be engaged by a SHGC from the radial artery and the PCI successfully completed in almost all cases, use of the SHGC was safe with no guide catheter dissection, significant vascular complications, forearm haematoma, or major bleeding, and there was a low of radial artery occlusion.

Avoiding the need for initial sheath placement prior to introduction of a sheathless guide catheter has the potential benefits of reducing cost, procedure time, and trauma to the radial artery. The SHGC system was developed to allow direct insertion. The 0.025″ angled Silverway wire was inserted through either a 20G needle or a 20G IV cannula in the radial artery successfully in all cases. Dilation was then performed with the short dilator with no skin incision being required in any of the cases. The SHGC with the long dilator inserted could be advanced into the aorta in all cases, with a low cross-over rate to femoral access when compared to other studies [1, 14]. There are a number of design features of the SHGC that may contribute its deliverability. The long tapered dilator inserted through the SHGC aids its introduction into the radial artery over a 0.025″ angiographic guide wire. The tapered shape of the dilator, the fact that there is no gap between the tip the dilator and the 0.025″ guide wire, and the smooth transition between the dilator and the tip of the SHGC along with the hydrophilic coating on the SHGC are all design features likely to facilitate delivery of the SHGC.

The principle benefit of using a sheathless guiding catheter during PCI is that the introducer sheath can be removed or avoided, minimising the external diameter of the equipment in the radial artery, while maintaining the internal diameter of the guide catheter. As such, the external diameter of a 6 or 7F SHGC is approximately 2F smaller than the external diameter of a conventional 6 or 7F radial sheath and approximately 1F smaller than thin wall radial sheaths such as the Glidesheath slender (Terumo Corp., Tokyo, Japan). Previous studies have clearly demonstrated an increasing incidence of radial artery occlusion with increasing sheath size [9, 15]. In our study, use of the SHGC was associated with a low rate of radial artery occlusion (1.6%) despite the frequent use of the 7F SHGC. This is consistent with previous studies using sheathless guide catheters which have also demonstrated low radial artery occlusion rates [16]. In addition, radial spasm is also more common when large sheaths and catheters are used in the radial artery [13]. In our study, there were no occurrences of significant radial artery spasm which is likely due to the combination of the reduced external diameter and the hydrophilic coating on the SHGC.

Despite the increase in use of radial artery access for PCI, many operators remain reluctant to use the radial approach for complex PCI often citing difficulty using large-bore guiding catheters and lack of support. Next to safety, efficacy is of paramount importance to guide access site selection in complex PCI. In our study, the SHGC was frequently used to treat complex lesions including calcified lesions, CTO, bifurcations, long lesions, and left main coronary lesions with a high success rate. The large inner lumen particularly of the 7F SHGC is compatible with a wide range of equipment and techniques. The 7 F SHGC can accommodate a rotational atherectomy burr up to 2.0 mm in size, IVUS with a low-profile microcatheter facilitating real-time visualisation and puncture of a proximal CTO cap, and a low-profile IVUS alongside a stent allowing real-time IVUS-guided ostial stent placement [17]. The combination of Henka Braid and Hyper Shaft technology and improved shapes in the SHGC has increased the manoeuvrability and backup when compared to the sheathless Eaucath. Despite the frequent use of 7F catheters with supportive shapes (AL, PB, and SPB), there were no guide catheter-related dissections in our study. The SHGC tip has been designed to reduce the risk of guide catheter-related trauma being composed of flexible urethane and tungsten powder and having rounded edges.

We noted the angled 0.025″ Silverway wire can go into side branches relatively easily. We recommend advancing under fluoroscopy if there is any resistance to advancement. The PB and SPB 3 and 3.5 curves are larger than EBU and XB 3 and 3.5 curves, respectively. This meant that the PB 3.5 and SPB 3.5 curves were often too large resulting in PB3 and SPB3 curves being more frequently used. In long cases, if the surface of the SHGC dried out, there was resistance to advancing the guide catheter further at the skin level in some cases. This could be resolved by moistening the catheter with a wet gauze.

Some limitations of our study must be acknowledged. The results in this study are from a single high-volume centre, with high-volume operators with extensive transradial experience and experience with use of sheathless guide catheters, which needs to be taken into account in interpreting the study results. We did not routinely use radial ultrasound to access radial patency postprocedure, and it is therefore possible that the rate of acute occlusion may be underestimated.

This first clinical experience with the SHGC demonstrates that direct insertion of the sheathless Hyperion catheter is safe and effective. Use of the SHGC allows complex interventions using a wide range of adjunct devices and techniques to be undertaken transradially with high success and low complication rates.

## Figures and Tables

**Table 1:** Patient demographic and clinical data.

Variable	*n* = 120
Age (years)	67.1 ± 12.6
Male sex (%)	95 (79.2)
Median BMI (kg/m^2^)	28.4 ± 7.7
Diabetes mellitus (%)	23 (19.2)
Hypertension (%)	75 (62.5)
Hyperlipidaemia (%)	96 (80)
Current smokers (%)	21 (17.5)
Previous MI (%)	46 (38.3)
Previous PCI (%)	47 (39.2)
Previous CABG (%)	8 (6.7)
Acute coronary syndrome (%)	55 (45.8)

BMI, body mass index; CABG, coronary artery bypass grafting; MI, myocardial infarction; PCI, percutaneous coronary intervention.

**Table 2:** Arterial access and catheter characteristics.

Variable	*n* = 128
Right radial	120 (93.8)
Left radial	8 (6.2)
Sheathless Hyperion size	
6F (%)	50 (39.1)
7F (%)	78 (60.9)
Insertion method	
Direct via 20G needle	59 (46.1)
Direct via IV cannula	15 (11.7)
Sheath then sheathless	24 (18.8)
Through sheath	30 (23.4)
Sheathless Hyperion shape	
PB 3.0 (%)	32 (25)
PB 3.5 (%)	25 (19.5)
SPB 3.0	15 (11.7)
SPB 3.5	4 (3.1)
JL 3.5	1 (0.8)
JR 4.0 (%)	18 (14.1)
AL 0.75 (%)	16 (12.5)
AL1 (%)	5 (3.9)
SAL1 (%)	11 (8.6)

IVUS, intravascular ultrasound; PCI, percutaneous coronary intervention.

**Table 3:** Lesion characteristics.

Variable	*n* = 150
Lesion location	
LMCA	7 (4.7)
Left anterior descending (%)	59 (39.3)
Circumflex (%)	30 (20)
Right coronary (%)	54 (36)
Lesion complexity	
Moderate or severe calcification (%)	52 (34.7)
Chronic total occlusion (%)	25 (16.7)
Bifurcation (%)	45 (30)
Long lesion (%)	68 (45.3)
Ostial lesion (%)	19 (12.7)
Intravascular imaging	125 (83.3)
Rotational atherectomy	17 (11.3)
Shockwave	19 (12.7)
Mean stent diameter (mm)	3.45 ± 0.59
Mean total stent length (mm)	34.6 ± 23.9
Mean postdilation balloon diameter	3.9 ± 0.71

CTO, chronic total occlusion; DES, drug-eluting stent.

## Data Availability

Access to individual patient data is restricted due to patient privacy. However, data are available from the corresponding author upon request.

## References

[1] Jolly S. S., Yusuf S., Cairns J. (2011). Radial versus femoral access for coronary angiography and intervention in patients with acute coronary syndromes (RIVAL): a randomised, parallel group, multicentre trial. *The Lancet*.

[2] Valgimigli M., Gagnor A., Calabró P. (2015). Radial versus femoral access in patients with acute coronary syndromes undergoing invasive management: a randomised multicentre trial. *The Lancet*.

[3] Valgimigli M., Frigoli E., Leonardi S. (2018). Radial versus femoral access and bivalirudin versus unfractionated heparin in invasively managed patients with acute coronary syndrome (MATRIX): final 1-year results of a multicentre, randomised controlled trial. *Lancet (London, England)*.

[4] Romagnoli E., Biondi-Zoccai G., Sciahbasi A. (2012). Radial versus femoral randomized investigation in ST-segment elevation acute coronary syndrome: the RIFLE-STEACS (radial versus femoral randomized investigation in ST-elevation acute coronary syndrome) study. *Journal of the American College of Cardiology*.

[5] Ferrante G., Rao S. V., Jüni P. (2016). Radial versus femoral access for coronary interventions across the entire spectrum of patients with coronary artery disease: a meta-analysis of randomized trials. *JACC: Cardiovascular Interventions*.

[6] Neumann F.-J., Sousa-Uva M., Ahlsson A. (2019). 2018 ESC/EACTS guidelines on myocardial revascularization. *European Heart Journal*.

[7] Mason P. J., Shah B., Tamis-Holland J. E. (2018). An update on radial artery access and best practices for transradial coronary angiography and intervention in acute coronary syndrome: a scientific statement from the American heart association. *Circulation. Cardiovascular Interventions*.

[8] Saito S., Ikei H., Hosokawa G., Tanaka S. (1999). Influence of the ratio between radial artery inner diameter and sheath outer diameter on radial artery flow after transradial coronary intervention. *Catheterization and Cardiovascular Interventions*.

[9] Bernat I., Aminian A., Pancholy S. (2019). Best practices for the prevention of radial artery occlusion after transradial diagnostic angiography and intervention: an international consensus paper. *JACC: Cardiovascular Interventions*.

[10] Thygesen K., Alpert J. S., Jaffe A. S. (2018). Fourth universal definition of myocardial infarction (2018). *Journal of the American College of Cardiology*.

[11] Moussa I. D., Klein L. W., Shah B. (2013). Consideration of a new definition of clinically relevant myocardial infarction after coronary revascularization: an expert consensus document from the Society for Cardiovascular Angiography and Interventions (SCAI). *Journal of the American College of Cardiology*.

[12] Cutlip D. E., Windecker S., Mehran R. (2007). Clinical end points in coronary stent trials: a case for standardized definitions. *Circulation*.

[13] Goldsmit A., Kiemeneij F., Gilchrist I. C. (2014). Radial artery spasm associated with transradial cardiovascular procedures: results from the RAS registry. *Catheterization and Cardiovascular Interventions*.

[14] Gragnano F., Branca M., Frigoli E. (2021). Access-site crossover in patients with acute coronary syndrome undergoing invasive management. *JACC: Cardiovascular Interventions*.

[15] Rashid M., Kwok C. S., Pancholy S. (2016). Radial artery occlusion after transradial interventions: a systematic review and meta-analysis. *Journal of American Heart Association*.

[16] Horie K., Tada N., Isawa T. (2018). A randomised comparison of incidence of radial artery occlusion and symptomatic radial artery spasm associated with elective transradial coronary intervention using 6.5 Fr sheathless eaucath guiding Catheter vs. 6.0 Fr Glidesheath Slender. *EuroIntervention*.

[17] Harding S. A., Webber B., Fairley S., Ormiston J. A. (2021). Real-time intravascular ultrasound guidance: a novel technique for accurate placement of ostial stents. *Catheterization and Cardiovascular Interventions*.

